# La patella cubiti découverte à la suite d’un traumatisme: à propos d’un cas et revue de la littérature

**DOI:** 10.11604/pamj.2019.32.128.16122

**Published:** 2019-03-18

**Authors:** Amour Espoir Mokoko-Louckou, Kevin Parfait Bienvenu Bouhelo-Pam, Badarou Chaibou, Ismail Abdouli, Mohammed Shimi, Abdelhalim El Ibrahim, Abdelmajid El Mrini

**Affiliations:** 1 Service de Traumato-orthopédie B4, CHU Hassan II, Fès, Maroc

**Keywords:** Coude, olécrane, anomalie congénitale, Elbow, olecranon, congenital anomaly

## Abstract

La patella cubiti est une anomalie très rare qui se caractérise par la non union de l'olécrane au cubitus proximal. Nous rapportons un patient de 27 ans admis pour traumatisme du coude dont le diagnostic révélait une patella cubiti faisant confondre avec une fracture de l'olécrane. Le patient a bénéficié d'une résection du fragment osseux. A 3 mois de l'intervention, le patient s'est senti satisfait car le corps étranger qu'il ressentait au niveau du coude a disparu.

## Abstract (en)

Patella cubiti is a very rare anomaly characterized by the non-union of olecranon and proximal ulna. We here report the case of a 27-year old patient admitted with a trauma of the elbow. Diagnostic examination revealed patella cubiti mimicking olecranon fracture. The patient underwent excision of bone fragment. Three months after surgery, the patient was satisfied because the foreign body that he felt at the level of the elbow had disappeared.

## Introduction

La non union congénitale de l'olécrane que Habbe a appelé «patella cubiti», est une variation anatomique très rare [1]. Dans un cas typique, l'olécrane entier ou une partie de celui-ci est séparé du cubitus proximal, les bords osseux sont réguliers et lisses avec un contour cortical de chaque côté. Généralement il est asymptomatique et son diagnostic est de découverte fortuite. Nous rapportons un patient dont le diagnostic est posé dans les suites d'un traumatisme.

## Patient et observation

**Figure 1:**
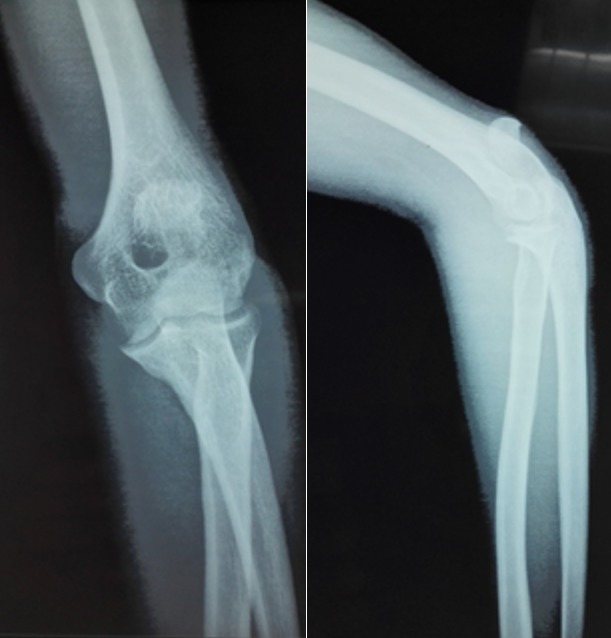
radiographie du coude de face et de profil

**Figure 2:**
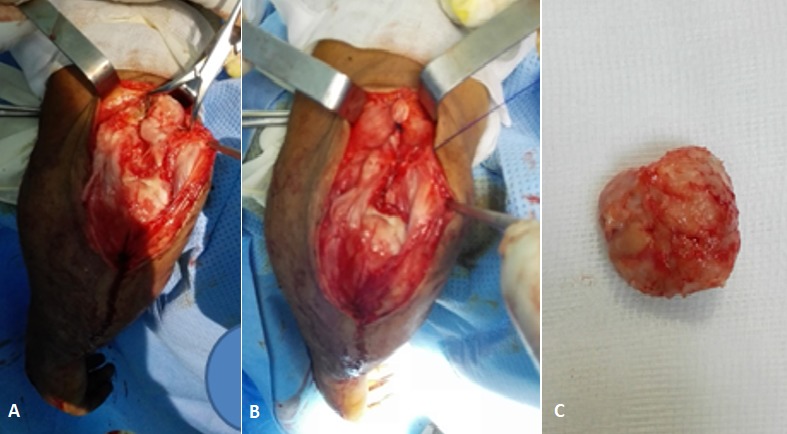
image per opératoire: A) mise en évidence de la patella cubiti; B) libération du fragment osseux dans le tendon tricipital; C) fragment osseux de la patella cubiti

Il s'est agi d'un patient de 23 ans, tabagique et alcoolique, sans profession, admis dans notre formation pour la prise en charge d'un traumatisme ouvert du coude gauche suite à une agression par coup de couteau. Avant le traumatisme, le patient palpait une masse ferme indolore à la face postérieure du coude gauche, individualisé de l'olécrane. Cette masse non symptomatique n'avait fait l'objet d'aucune exploration. A l'examen physique du coude gauche, on a noté une plaie d'environ 3 cm de la face postéro-externe du coude, pas d'atteinte vasculo-nerveuse, ni atteinte de la mobilité du coude. La radiographie du coude gauche de face et de profil a montré un fragment osseux à bord régulier en regard de l'olécrane (Figure 1). Le diagnostic de fracture de l'olécrane est posé et une chirurgie est indiquée. Le patient était installé au bloc en décubitus latéral et bras sur pose bras. Nous avons élargi la plaie de telle sorte qu'on reproduise l'abord postérieur du coude. En peropératoire, nous avons retrouvé un tendon tricipital intact avec un fragment osseux arrondi régulier à environ 3 cm du bec de l'olécrane encastré dans ce tendon rappelant l'anatomie de la patella (Figure 2). Ce fragment osseux a été réséqué sur demande du patient. A 6 mois de recul, le patient ne s'est pas plaint d'un changement sur la fonctionnalité du coude.

## Discussion

En 1903, Kienbock rapporta qu'un homme avait un gros os sésamoïde ressemblant à une patella près de l'olécrane [2]. La majorité des cas sont signalés chez les jeunes de sexe masculin, mais quelques cas précis de patella cubiti sont signalés chez les sujets de sexe féminin [3]. Un fragment osseux unilatéral ou bilatéral au sein du tendon du triceps est appelé une patella cubiti; cette condition est rarement rencontrée et plusieurs théories concernant son étiologie ont été proposées: séparation de l’épiphyse du centre de l’olécrâne dans la petite enfance, menant au développement indépendant de ce centre [4]. Dans notre étude, l'étiologie n'est pas évidente car on n'a pas retrouvé une notion de traumatisme en amont du traumatisme. Notre revue de la littérature permet d'avoir des données pour une meilleure compréhension de la patella cubiti. Bien que le traitement de la patella cubiti reste controverse, notre suggestion est d’éviter une intervention chirurgicale que chez les patients qui présentent uniquement une raideur du coude [5].

## Conclusion

Les accidents traumatiques au coude sont fréquents dans l’enfance mais les radiographies sont rarement faites; pour cette raison des anomalies squelettiques mineures ne sont souvent pas détectées. Ces enfants peuvent présenter plus tard une “patella cubiti”. Mais l'origine congénitale est retenue en absence de preuve étiologique et la vie peut être avec une “patella cubiti”.

## Conflits d’intérêts

Les auteurs ne déclarent aucun conflits d'intérêts.
